# Serum and Cerebrospinal Fluid Levels of S-100β Is A Biomarker for Spinal Cord Injury; a Systematic Review and Meta-Analysis

**Published:** 2019-02-12

**Authors:** Gholamreza Faridaalee, Fatemeh Keyghobadi Khajeh

**Affiliations:** 1 Department of Emergency, Maragheh University of Medical Sciences, Maragheh, Iran; 2 Department of Community Medicine, Tabriz University of Medical Sciences, Tabriz, Iran

**Keywords:** S100 Calcium Binding Protein beta Subunit, Spinal Cord Injuries, Animals, S100b protein, rat

## Abstract

**Introduction:**

There is controversy regarding the value of serum or cerebrospinal fluid (CSF) levels of S100 calcium-binding protein B (S-100B) in spinal cord injury (SCI). For reaching a general conclusion, the present meta-analysis was designed aiming to evaluate the value of serum and CSF levels of S-100B protein in detecting the presence of SCI in animal studies.

**Methods:**

An extensive search was performed in Medline, Embase, Scopus and Web of science databases. Screening articles, summarizing them and entering data to checklist and quality assessment of the mentioned articles were done by 2 independent reviewers. Data were analyzed and a pooled standardized mean difference (SMD) and 95% confidence interval (95% CI) were presented.

**Results:**

Finally, the data of 7 articles were included in the meta-analysis. Serum level of S-100B had increased as a result of SCI. During the first 6 hours after injury, the level of this protein was very high (SMD=3.8; 95% CI: 2.6 to 5.1; p<0.0001), but as time passed the serum level of the protein had decreased (SMD=0.4; 95% CI: -1.2 to 2.0; p=0.65). In addition, CSF level of the mentioned protein was very high during the initial 6 hours after injury (SMD: 5.8; 95% CI: 3.6 to 8.0), and this elevated level was still observed until 12 hours after injury (SMD: 6.5; 95% CI: 3.7 to 9.3; p<0.0001).

**Conclusion:**

The results of the present systematic review and meta-analysis show that measuring the level of S-100Β protein in serum and CSF has a potential value in diagnosis of SCI in animal models. This biomarker increases during the initial 6 hours following injury and remains high until 24 hours after that. However, more than 24 hours after the injury, serum level of this protein returns to the level of animals without SCI.

## Introduction:

Traumatic spinal cord injury (SCI) is among the most serious injuries that deeply affect the health of an individual. Prevalence of SCI has been reported as 11 to 53 cases for each million population (1). Epidemiologic studies performed in the past 3 decades have clearly shown that SCIs primarily affect young individuals (with the mean age of 29 years) and then impact the 30-45 years age group (2-4). In all age groups, the highest rate of spinal cord injury belongs to incomplete tetraplegia, and after that, complete paraplegia, complete tetraplegia, and incomplete paraplegia are the most common, respectively (5). Despite extensive research in the field of SCIs no effective treatment has been found for restoring motor and sensory functions, yet (6), but considerable advances in looking after and providing care for SCI patients has led to a significant decrease in the rate of mortality due to SCI (7). 

After stabilizing the clinical condition in the initial days after spinal cord injury, the family of the patients and the patients themselves want to know if they can walk again or if they will be able to carry out their personal obligations such as eating, taking a bath, and wearing clothes or not (8); therefore, a correct evaluation of the severity and classification of SCI for predicting the functional status after spinal cord injury is of importance. Currently, classification of SCIs is done based on American Spinal Injury Association (ASIA) Impairment Scale (AIS) (9). Although AIS is currently a gold standard in classification of SCI, this system has some limitations too (10). Therefore, in order to create a more comprehensive classification, the researchers have tried to use various tools such as magnetic resonance imaging (MRI) (11-13), electrophysiological evaluations (14-16), and biomarkers (17, 18). Biomarkers are secreted to the serum or cerebrospinal fluid (CSF) at various stages and in different types of SCI. One of the biomarkers, which has received much attention in prediction of presence and severity of the injury is S-100Β protein, as studies have shown a rapid increase in its serum level after spinal cord injury (19, 20). Yet, there is still no data regarding optimum timing of measuring this protein or the effect of injury severity on its serum or CSF level. For reaching a general conclusion, the present study was designed aiming to evaluate the diagnostic value of serum and CSF levels of S-100Β protein in detecting the presence of SCI in animal studies.

## Methods:

The present study was designed based on MOOSE guideline, which is a guide for performing systematic review and meta-analysis on observational studies (21). Defining PICO in the present study is as follows:

The problem or the study population includes animals with SCI; the intended factor (index test): the level of S-100Β protein in serum or cerebrospinal fluid; comparisons (C): comparison is done with a control group free of injury; and the studied outcome (O) includes the severity of injury and presence or absence of SCI.

***Search strategy***

For reaching the aims of the present study, an extensive search was performed in the electronic databases and references of related articles. Search in grey literature is another strategy used in the present study. Search in electronic databases was performed using the systematic method under the guidance of a librarian and supervision of an expert in the field of SCI. At this stage, related keywords were selected using MeSH and Emtree databases, consulting with experts in this regard, and searching in the titles and abstracts of related articles. Then search strategy for each database was defined using the guidelines of the same database. Methods of search and summarizing data have been reported in previous meta-analyses (22-34). It should be noted that electronic databases of Medline, Embase, Web of Science, and Scopus were searched until the end of 2017. Search strategy in Medline database is presented below as a template.

Selection criteria

In the present research, experimental studies performed with the aim of determining the diagnostic accuracy of serum and cerebrospinal fluid levels of S-100Β protein in detecting spinal cord injury were included. Only the studies that had a control group were included. Exclusion criteria consisted of absence of a control group, not reporting the protocol of measuring the biomarker and review articles.

Quality assessment and Data Extraction

Screening articles, summarizing them and entering data to checklist and quality assessment of the mentioned articles were done by 2 independent individuals. Any disagreement was resolved via discussion with a third researcher. The articles were summarized using a checklist that was designed based on the guidelines of PRISMA statement (35). Extracted data included information regarding study design, characteristics of case and control groups (age, mechanism of spinal cord injury induction), the number of studied cases, and serum and CSF levels of S-100Β protein. If 2 or more articles were published from the same dataset, the study which had the biggest sample size or the longest follow up was included. If the required data were not presented in the paper, the corresponding author was contacted and asked for the required data. When the evaluated variables were presented based on various subgroups (such as sex and etc.), data were recorded separately. If the results were given as charts, the method of data extraction from charts introduced by Sistrom and Mergo was used (36).

Quality control of the study

The quality was assessed using the criteria proposed by Yousefifard et al. (37) and Hassannejad et al. (38). For assessing the agreement between the 2 researchers, inter rater reliability was evaluated in quality assessment of the studies (agreement rate: 88%). In case of any disagreement, it was resolved by discussion with a third researcher.

Statistical analyses

Analyses were done using STATA 14.0 statistical software. All studies were summarized and classified based on the studied variables. In the mentioned statistical software, analyses were done using the “metan” command and forest plots of serum and CSF levels of S-100Β protein in detection of spinal cord injury were drawn. In the present research, depending on the presence or absence of heterogeneity, random effect model or fixed effect model were used, respectively, for performing analyses. For evaluating heterogeneity between the studies, chi square and I^2^ tests were applied. In cases that heterogeneity was present, subgroup analyses were performed to determine the cause of heterogeneity.

## Results:

- ***Study characteristics***

**Figure 1:**
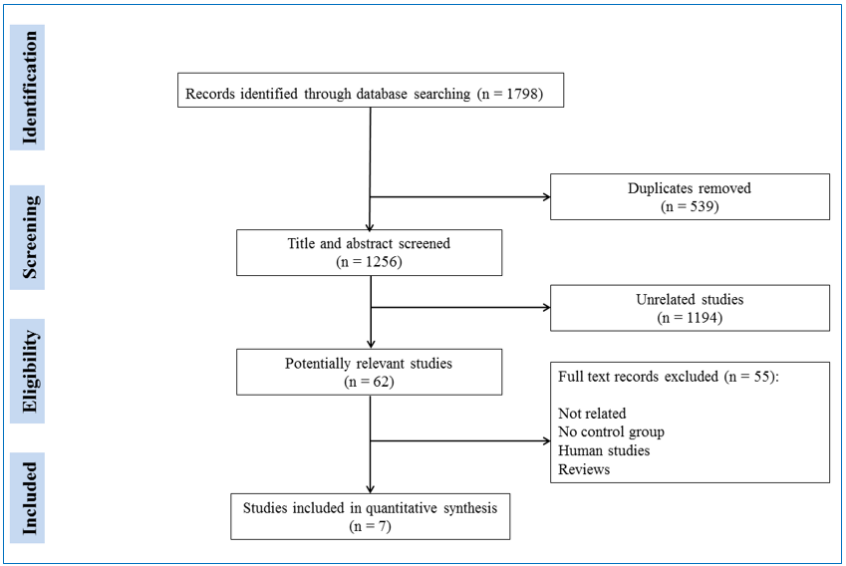
PRISMA flow diagram of the present meta-analysis

The search performed in databases yielded 1798 non-redundant records. After screening, finally the data of 7 articles were included in the meta-analysis (39-45) (Figure 1). These studies consisted of 136 healthy animals and 128 animals with SCI. 6 studies were performed on rats (39-44) and only 1 study was performed on pigs (45). Injury severity was moderate to severe. The model of spinal cord injury used was contusion in 4 studies (39-41, 44), compression in 2 studies (42, 43), and Armor blunt trauma in one study (45). Time of sampling and evaluation of S-100Β protein varied from 30 minutes to 240 hours. For performing analyses, time to sample was classified into 4 groups of 0 to 6 hours after injury, 12 hours after injury, 24 hours after injury and more than 24 hours after injury. It should be noted that 4 studies had assessed serum levels of S-100Β protein (40-43), one study had evaluated CSF level of this protein (39) and two had evaluated both (44, 45). Summary of the mentioned variables are reported in table 1.

- ***Source of bias***

**Figure 2:**
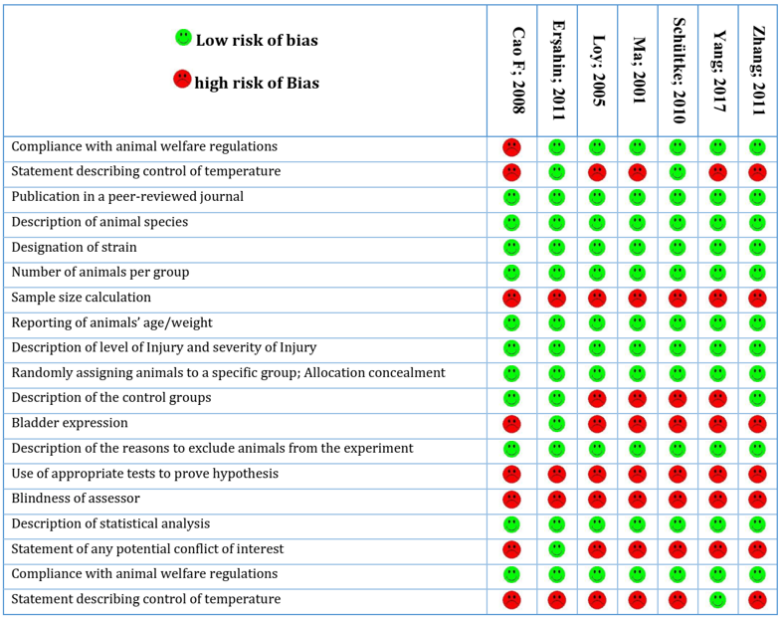
Quality assessment of the included studies

In quality control of the studies, the method suggested by Hassannejad et al. and Yousefifard et al. was applied. Findings of this section have been presented in figure 2. As can be seen, no study had attempted to calculate sample size, none had presented findings regarding the mortality of the animals, and quality of the studies regarding post-operative care of the animals was poor. It should be noted that conflict of interest was reported in only one study. Out of the 19 items being evaluated in the quality assessment of the articles, 11 items were desirable in almost all studies.

Heterogeneity test showed that in evaluating both the diagnostic value of serum level (I^2^=86.3; p<0.0001) and diagnostic value of CSF level (I^2^=79.5; p<0.0001) of S-100Β protein, significant heterogeneity was present. Therefore, subgroup analysis was performed.

Meta-analysis

- ***Serum value of S-100Β protein in detection of SCI***

**Figure 3:**
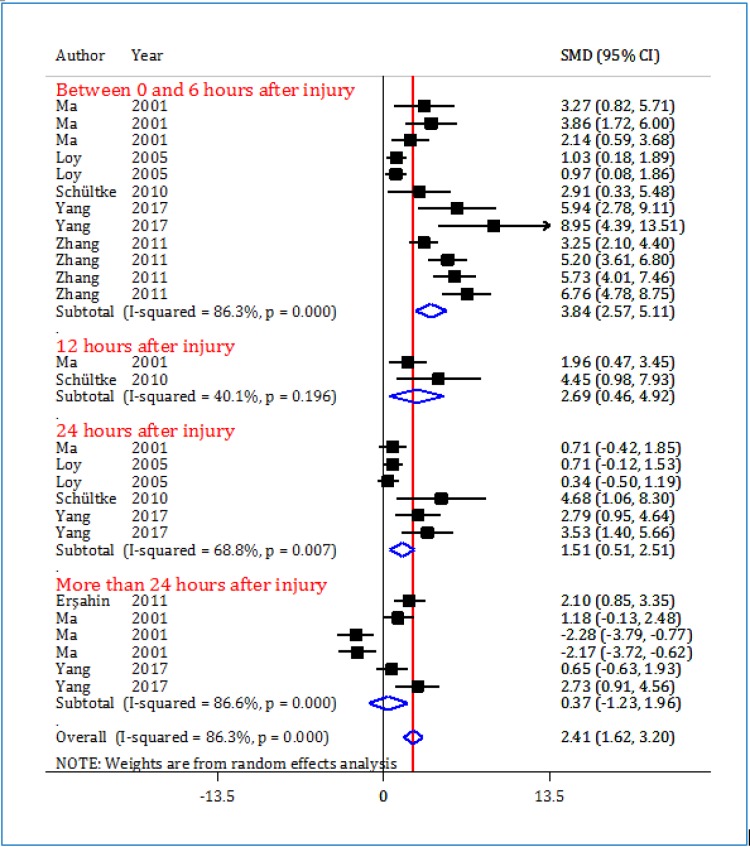
Forest plot of serum S-100B in spinal cord injury. Animal studies showed that the mean level of serum S-100B is higher in animals with spinal cord injury during the first 24 hours after trauma. CI: Confidence interval; SMD: Standardized mean difference

The findings showed that SCI can be detected via serum level of S-100Β protein. In other words, serum level of this protein increases as a result of SCI (figure 3). During the first 6 hours after injury, the level of this protein was very high (SMD=3.8; 95% CI: 2.6 to 5.1; p<0.0001), but as time passed the serum level of the protein had decreased and after more than 24 hours had passed, its measure was almost the same as the animals without a spinal cord injury (SMD=0.4; 95% CI: -1.2 to 2.0; p=0.65).

It should be noted that the serum level of this protein in severe injuries (SMD=3.4; 95% CI: 1.6 to 5.4; p<0.0001) was many times more than moderate injuries (SMD=1.6; 95% CI: 0.8 to 2.4; p<0.0001) (p=0.04) (table 2).

- ***CSF value of S-100Β protein in detection of SCI***

**Figure 4:**
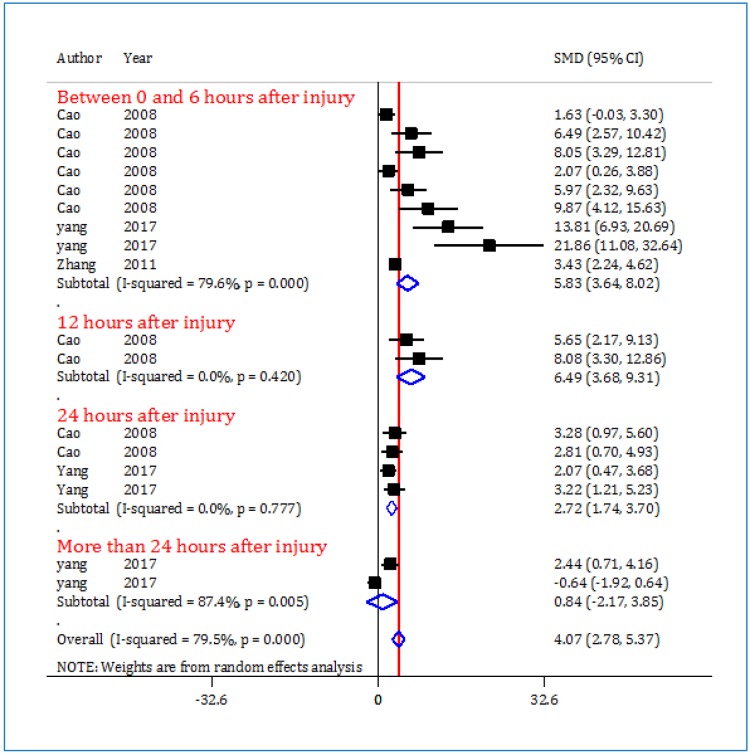
Forest plot of CSF S-100B in spinal cord injury. Animal studies showed that the mean level of CSF S-100B is higher in animals with spinal cord injury during the first 24 hours after trauma. CI: Confidence interval; SMD: Standardized mean difference

Just like the serum level, CSF level of S-100Β protein had significantly increased following spinal cord injury. CSF level of the mentioned protein was very high (SMD: 5.8; 95% CI: 3.6 to 8.0), and this increased level was still observed until 12 hours after injury (SMD: 6.5; 95% CI: 3.7 to 9.3; p<0.0001). However, 24 hours after injury this rate had decreased (SMD: 2.7; 95% CI: 1.7 to 3.7; p<0.0001) and after more than 24 hours, CSF level of this protein in animals with SCI was not different from the healthy animals group (SMD: 0.7; 95% CI: -2.2 to 3.8; p=0.584) (Figure 4). 

## Discussion:

Most studies in the field of biomarkers related to SCI are performed on NSE and S-100Β, but since these two biomarkers have low specificity in patients who have multiple traumas simultaneously (18) (these biomarkers also increase in traumas other than SCI), performing a systematic review seemed necessary for reaching a definite conclusion regarding the effectiveness of these biomarkers in detection of SCI; therefore, the present systematic review evaluated the diagnostic value of serum and cerebrospinal fluid levels of S-100Β protein in detection of SCI for the first time. The results of this study show that S-100Β protein levels in serum and CSF increase in animals following SCI induction and have diagnostic value. During the initial 6 hours of SCI, the level of this protein is very high in CSF and serum, but with time passing, the serum level of this protein decreases and at times after 24 hours, its rate does not differ from animals without SCI. 

**Table 1:** Characteristics of included studies

**Author; year; country**	**Species**	Sample size **(no-SCI / SCI)**	**Weight**	**Severity**	**SCI-model**	**Time to sample (hours)**	**Method of S-100β analysis**	**Location of biomarker**
Cao F; 2008; China (39)	Sprague-Dawley rat	40 / 40	200 gr	Moderate to severe	Contusion	0.5 to 24	ELISA	CSF
Erşahin; 2011; Turkey (40)	Wistar albino rat	8 / 8	250-300 gr	Moderate	Contusion	168	ELISA	Serum
Ma; 2001; Sweden (42)	Sprague-Dawley rat	48 / 40	200-300 gr	Moderate	Compression	0 to 240	ELISA	Serum
Loy; 2005; USA (41)	Sprague-Dawley rat	12 / 12	190-230 gr	Moderate to severe	Contusion	6 to 24	ELISA	Serum
Schültke; 2010; Canada (43)	Wistar rat	9 / 9	286-310 gr	Moderate	Compression	6 to 24	ELISA	Serum
Yang; 2017; USA (44)	Fischer rat	5 / 5	220-250 gr	Moderate to severe	Contusion	4 to 68	ELISA	CSF and serum
Zhang; 2011; China (45)	White pig	14 / 14	41.5-61 kg	Severe	Armor Blunt Trauma	0.5 to 3	ELISA	CSF and serum

CSF: Cerebrospinal fluid; ELISA: Enzyme-linked immunosorbent assay

**Table 2:** Subgroup analysis of S-100Β level in traumatic spinal cord injury

**Variable**	**Heterogeneity**	**P for heterogeneity**	**Effect size**	**P**
**Serum level of S-100Β**				
**Severity of injury**				
Moderate	78.5%	<0.0001	1.6 (0.8 to 2.4)	<0.0001
Severe	91.0%	<0.0001	3.4 (1.6 to 5.4)	<0.0001
			*Significance level between groups*	**0.040**
**Injury model**				
Contusion	73.9%	<0.0001	1.8 (1.0 to 2.6)	<0.0001
Compression	82.8%	<0.0001	1.6 (0.3 to 2.9)	<0.0001
			*Significance level between groups*	0.122
**Time to sample ** **after injury**				
0 to 6 hours	86.3%	<0.0001	3.8 (2.6 to 5.1)	<0.0001
12 hours	40.1%	0.196	2.7 (0.5 to 4.9)	0.018
24 hours	68.8%	0.007	1.5 (0.5 to 2.5)	0.003
More than 24 hours	86.6%	<0.0001	0.4 (-1.2 to 2.0)	0.652
			*Significance level between groups*	**0.003**
**CSF level of S-100Β**				
**Severity of injury**				
Moderate	70.1%	<0.0001	4.1 (2.4 to 5.8)	<0.0001
Severe	85.1%	<0.0001	4.1 (2.1 to 6.2)	<0.0001
			*Significance level between groups*	0.925
**Time to sample** ** after injury**				
0 to 6 hours	79.6%	<0.0001	5.8 (3.6 to 8.0)	<0.0001
12 hours	0.0%	0.420	6.5 (3.7 to 9.3)	<0.0001
24 hours	0.0%	0.777	2.7 (1.7 to 3.7)	<0.0001
More than 24 hours	87.4%	0.005	0.8 (-2.2 to 3.8)	0.584
			Significan*ce *level between groups	0.051

S-100Β protein, which is a calcium-binding protein, is mostly present in the cytoplasm of glial cells. Since the blood-brain barrier (BBB) is not permeable to this protein, the measure of this protein in serum and CSF is normally zero and therefore, following injury to the central nervous system and damage of BBB, the level of this biomarker increases in CSF and serum depending on the severity of injury (46, 47). The present systematic review has evaluated the level of S-100 protein in animal models of SCI. In the systematic review performed by Salehpoor et al. in 2015, the level of various biomarkers including S-100 has been evaluated in traumatic brain injuries (TBIs) in clinical studies and it has been shown that the serum level of S-100 in children and adults strongly correlates with TBI diagnosis and prediction of its outcome (48). The systematic review by Thelin et al. in 2017 showed that serum level of biomarkers such as S-100 is effective in monitoring brain injuries in adults (49). A systematic review by Lugones et al. in 2018 presented the same results in children (50). Since spinal cord is a part of the central nervous system just like the brain and has BBB, the results of our study can also be in line with the existing studies and damage to BBB following SCI can be a logical explanation for the results of our study.

Various methods such as standard scoring system, magnetic resonance imaging (MRI), and electro-physiologic techniques are used for detection and classification of SCI. With the invention of diagnostic methods with high accuracy, such as enzyme-linked immunosorbent assay (ELISA), immunoblotting, proteomics and genomics, one diagnostic method for SCI is evaluating the level of biomarkers in blood and CSF (51). The most famous study in the field of assessing the correlation between biomarkers and diagnosis of SCI might be the study by Guez et al. in 2003. This research team proposed and evaluated the idea of assessing the level of biomarkers in CSF as a diagnostic tool for SCI (52). In that study, the level of NFL and GFAP was evaluated in CSF of patients with acute SCI and it was revealed that measuring these biomarkers in CSF can be used as a tool for quantitative classification of injured neurons following various degrees of SCI. In 2010, for the first time, in addition to CSF, these biomarkers were measured and assessed in blood of patients with various SCI severities and with acceptable sample size by Kwon et al. The results of the study expressed that measurement of IL-8, S-100B, and GFAP in CSF during the initial 24 hours following SCI is effective in determining the severity of injury and monitoring improvement process (53). Kwon et al. also extensively assessed the value of measuring biomarkers in CSF and serum following SCI in a review in 2011 and finally stated that considering the scarcity of studies to date, reaching a final conclusion regarding the value of measuring biomarkers in serum and CSF for classification of SCI severity and monitoring of the improvement process is not possible yet (17). In addition to their diagnostic value, biomarkers are also useful in choosing a strategy for selecting a treatment plan in SCI. Since the outcomes of the primary phase of SCI are unavoidable, the main goal of treatment in SCI is preventing the secondary phase during which many things happen on the molecular level and the level of neural biomarkers is extremely mutable. Therefore, these biomarkers can be studied in the second phase for following the interventions performed (54, 55).

Limitations

High level of heterogeneity was among the limitations of the present study. One of the sources of the high heterogeneity was the time of measuring serum and CSF levels of this protein. However, other factors such as difference in techniques used for evaluating the level of S-100Β, difference between various species and etc. might be among other factors causing heterogeneity. In this study, all efforts were made to also include clinical studies that had evaluated the diagnostic value of S-100Β in diagnosis of SCI. Yet, due to the small number of these studies, various methodologies for performing the study and a high level of diversity in the studied SCI patient population, this could not be done.

## Conclusion:

The results of the present systematic review and meta-analysis show that measuring the level of S-100Β protein in serum and CSF has diagnostic value in diagnosis of SCI in animal models. This biomarker increases during the initial 6 hours following injury and remains high until 24 hours after it. However, more than 24 hours after the injury, serum level of this protein returns to the level of animals without SCI.
